# A comparison of E15.5 fetus and newborn rat serum proteomes

**DOI:** 10.1186/1477-5956-10-64

**Published:** 2012-11-07

**Authors:** Lilong Wei, Lulu Jia, Lisi Zhu, Sucan Ma, Dan Zhang, Chen Shao, Wei Sun, Youhe Gao

**Affiliations:** 1 Department of Physiology and Pathophysiology, National Key Laboratory of Medical Molecular Biology Institute of Basic Medical Sciences, Chinese Academy of Medical Sciences, Peking Union Medical College, Beijing, 100005, China; 2 Department of Core Instrument Facility, Institute of Basic Medical Sciences, Chinese Academy of Medical Sciences, Peking Union Medical College, Beijing, 100005, China

**Keywords:** Serum proteome, Development, Littermate variation

## Abstract

**Background:**

Serum proteins carry out several functions in the circulation, including transfer, immunological functions, messenger functions, coagulation, and regulation of homeostasis. To investigate changes in serum proteins that occur during development, the serum proteomes of embryonic 15.5 (E15.5) fetuses and newborn rats were compared using LC-MS/MS.

**Results:**

A total of 958 proteins were identified in the serum of rats at both developmental stages. The serum proteome pattern of newborn rats was compared to E15.5 fetuses by relative quantitation. The expression patterns of hemoglobin subunits were different at the two stages, with most of the subunits having decreased expression in newborn rats compared to E15.5 fetuses. In addition, 8 of 12 apolipoproteins were significantly decreased and 10 of 11 identified complement molecules were increased, with 4 exhibiting a significant increase. Moreover, 11 of 14 of the significantly increased enzyme regulators were inhibitors. The serum proteome patterns of different littermates from both developmental stages were also compared. We found that the levels of many highly abundant serum proteins varied between littermates, and the variations were larger than the variations of the technical control.

**Conclusions:**

The serum proteomes of newborn rats and E15.5 fetuses were compared. The expression patterns of hemoglobin subunits were different at the two developmental stages, with most of the subunits having decreased expression. The majority of apolipoproteins had significantly decreased expression, while almost all identified complement proteins had increased expression. The levels of several highly abundant serum proteins also varied among littermates at these two developmental stages. This is the first study using LC-MS/MS to investigate serum proteome development.

## Background

Plasma, which is the soluble component of blood, is the most complex human-derived proteome [1]. As blood flows through tissues and organs of the human body, almost every cell in the body can communicate with plasma directly or indirectly and release a portion of their content into plasma through active secretion or leakage [2,3]. Serum consists of blood plasma without fibrinogens and includes all proteins not used for blood coagulation. Therefore, plasma and serum contain extremely informative proteomes that may contain unique information from different tissues and organs in the body. Plasma had been used to monitor the health status of patients by clinicians for many years [4], and it is thought that one plasma/serum proteome corresponds to a unique description of a patient experiencing a specific disease or physiological state.

Embryonic development is a complicated biological process whereby many rapid changes occur. Morphological changes that occur in the embryo have been well-documented in both rat and mouse animal models [5,6]. During the course of embryonic development, each organ of the body performs diverse biological processes and coordinates to form an extremely intricate life process. The composition of the serum proteome can change during embryonic development. Therefore, delineation of the molecular events involved in different stages of the serum proteome would not only advance our knowledge about the development of serum, but also of the entire body. Comparing the plasma proteome during the development process may help us identify markers that can be used to determine the different stages of body development [7].

Before the era of proteomics, changes in the protein composition during plasma and serum development were studied using paper electrophoresis or immune-electrophoresis in rat [8-11], mouse [12], chick [13-16], sheep [17], goat [18], pig [15], and human [19,20]. Using these methods, the patterns of highly abundant plasma and serum proteins, including albumin, globulin, transferrin, and alpha-fetoprotein (AFP), were described. One study investigated a total of 16 proteins using serum or cultured tissues obtained from human embryos and fetuses, and some proteins were found to be related to organ development [21]. Patterns of plasma and serum proteins in human fetuses and infants have been studied by high-resolution two-dimensional electrophoresis, and many proteins were identified, including AFP, which was found to progressively decrease during development [22]. However, to date, no methods based on liquid chromatography coupled with tandem mass spectrometry (LC-MS/MS) have been used to qualify and quantify proteins in serum at different development stages.

Individual variations exist ubiquitously throughout the world, including variations in body development. Therefore, it is important to delineate normal protein variations among individuals. The diversity of 25 proteins in human plasma was previously investigated using affinity-based mass spectrometry approaches [23]. Limited studies have also been performed in animal models.

This study investigated changes in serum functions during fetal development by comparing serum proteomes of embryonic day 15.5 (E15.5) fetuses and newborn rats. The quantitative characteristics of the serum proteomes were examined. Individual variations among littermates were also investigated at the proteome level. This study is the first to analyze serum changes between E15.5 fetuses and newborn rats using proteomic methodologies. In addition, the results may provide clues for understanding serum protein functions in future studies.

## Results and discussions

### Comparison of protein patterns in serum from E15.5 fetuses and newborn rats

#### SDS-PAGE analysis of serum proteins from E15.5 fetuses and newborn rats

**Figure 1:**
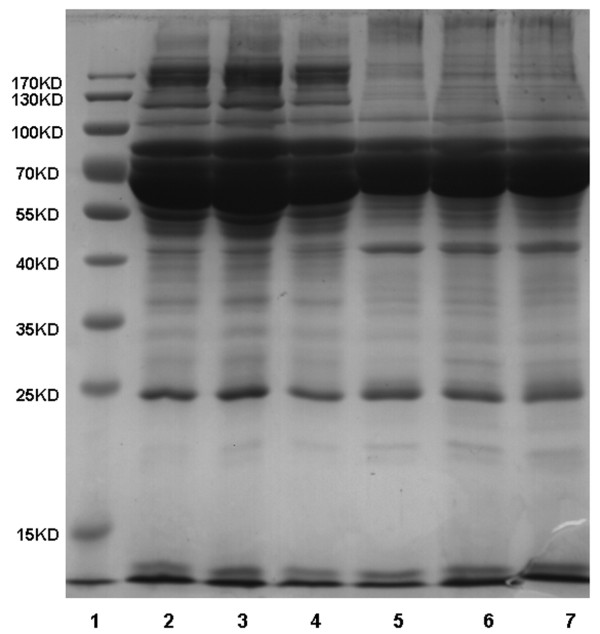
**Comparison of the protein pattern between E15.5 fetuses and newborn rats using 1D SDS-PAGE.** Twenty micrograms serum proteins from each individual E15.5 fetus or newborn rat were separated on a 1D SDS gel. The gel was stained with Coomassie Brilliant Blue. Lanes 2–4 indicate different individual newborn rats and lanes 5–7 indicate different individual fetuses.

The protein patterns of serum samples from E15.5 fetuses and newborn rats were first analyzed by SDS-PAGE. As shown in Figure 1, the protein patterns among different individuals were similar, while the patterns between the two development stages were different, even on SDS-PAGE.

### Changes in the serum proteomes of E15.5 fetuses and newborn rats based on LC-MS/MS analysis

Three individual samples each from E15.5 fetuses and newborn rats were identified using one dimensional (1D) LC-MS/MS. In total, 958 proteins were identified in all six MS runs (Additional file 1: Table S1). Using a two-tailed t-test for the samples from E15.5 fetuses and newborn rats, 47 proteins were found to be significantly increased and 57 were significantly decreased in newborn rats compared to E15.5 fetuses (p < 0.05) (Additional file 1: Table S1). In our study, individual samples, rather than mixture of the samples, were used to compare relative quantitation between newborn rats and E15.5 fetuses. Therefore, the changes in the proteomes between E15.5 fetuses and newborn rats were more likely caused by the true differences of the two stages during development because both individual and technical variations were considered.

It is better to analyze more samples. Since this is the first study that attempted to identify as many differentially expressed proteins as possible between E15.5 rat fetuses and newborns, profiling-based proteomic technology were used to identify the serum proteomes of the two stages. This technology is powerful for identifying large numbers of proteins in one experiment; however, it has very low efficiency with a limited sample throughput because of the time involved and high cost. Although the sample throughput of target proteomics has been improving, it requires knowledge from comprehensive profiling results. It can only quantify a certain number of proteins in one experiment and cannot identify as many differential proteins as we accomplished in profiling analysis. This study provided the foundation of a new research area and provided information for interested laboratories. Additional experiments were planned to confirm the findings.

Almost all proteins previously identified in the literature using electrophoresis, radio-electrophoresis, or two-dimensional (2D) electrophoresis in fetal plasma or serum from rat [11], chicken [13], pig [24-27], and human [22] were included in the 958 proteins identified in this study, with the exception of antithrombin III [22], which was not identified in our analysis. This discrepancy might be due to blood coagulation during sample processing. The changes observed for almost all of the proteins were consistent with published results in rat [11], chicken [13], pig [24-27], and human [22], such as Albumin, AFP, Complement 3, plasminogen, Alpha-2-Macroglobulin, Transferrin, and Alpha-1-acid Glycoprotein (Table 1). Apolipoproteins and hemopexin were found to be decreased in our analysis, which was opposite to that found in a study of the late gestation of the human fetus [21]. The reason for this discrepancy is currently not clear.

**Table 1:** Results from our study compared to results obtained prior to the modern era of proteomics

***Protein name***	***Our result***	***Published result***	***Species and reference***
Serum albumin	↑	↑	Rat [11], Pig [24], Chicken [13], Human [22], Porcine [27]
Isoform 1 of Serotransferrin	↑	↑	Rat [11] Human [21,22], Pig [25]
		∧	Porcine [27]
		↓	Pig [24]
Complement C3	↑	↑	Human [21]
		↓	Pig [24]
Alpha-2-macroglobulin	↑	↑	Human [21]
Plasminogen	↑	↑	Human [21,22]
		↓	Pig [24]
Isoform 1 of Haptoglobin	↑	#	Human [21]
Alpha-2-HS-glycoprotein	↑	↑	Human [22]
Transthyretin	↑	↑	Human [22]
Fetuin-B	↑	↑	Porcine [27],
		↑ or ↓	Pig [24]
Retinol-binding protein 4	↑	↑	Human [22]
Ceruloplasmin	↑	#	Human [21]
Fibrinogen-like 2	↑	#	Human [21]
Alpha-1-acid glycoprotein	↑	↑	Pig [24], Porcine [27]
Apolipoprotein H	↑	↑	Pig [24]
Angiotensinogen	↑	↓	Pig [24]
Hemopexin	↓	↑	Human [21]
Apolipoprotein A-I	↓	↑	Human [22]
Apolipoprotein E	↓	↑	Human [22]
Apolipoprotein A-IV	↓	↑	Human [22]
Gamma-A of Fibrinogen gamma chain	↓	#	Human [21]
Isoform 1 of Alpha-fetoprotein	↓	↓	Human [22], Pig [24,25],
		∧	Porcine [27]

Note: Proteins changed from E15.5 fetuses to newborn rats and proteins changed with development in literature were noted using an increasing arrow (↑) / decreasing arrow (↓). Proteins without information on expression changes are indicated with “#”. Protein expression that increased during the early stage of development and decreases in the late stage of development is indicated with “∧”.

**Figure 2:**
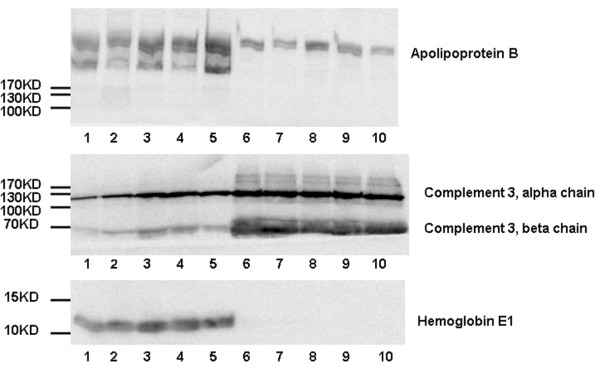
**Validations of C3, HBE1, and Apolipoprotein B by Western blot.** Western blot was performed to validate the changes in C3, HBE1, and Apolipoprotein B in five E15.5 fetus serum samples and five newborn rat serum samples. Thirty micrograms proteins of each specimen were loaded per lane. Lanes 1–5 indicate different E15.5 fetuses and lanes 6–10 indicate different newborn rats.

To confirm the differential proteins detected by mass spectrometry, Complement 3, Hemoglobin E1, and Apolipoprotein B were chosen for western blot analysis. As shown in Figure 2, the densitometries of the bands between two stages of development were calculated for different individuals, respectively (Figure 2 and Additional file 2: Table S2). Importantly the trends of changes were consistent with the trends found based on mass spectrometry data for all the different individuals.

### Comparison of hemoglobins

Eight hemoglobins or subunits were identified (Table 2). Hemoglobin zeta, beta 1, gamma 1, and epsilon 1 were significantly decreased and zero beta-1 globin was significantly increased in serum from newborn rats compared to serum from E15.5 fetuses (p < 0.05). Changes in these protein levels correlated well with the changes in gene expression during development in previously studies [28]. It has been reported that the epsilon globin gene is activated during the embryonic stage, the gamma globin gene is activated during the fetal period, and the beta globin gene is activated during the adult stage [28].

**Table 2:** Hemoglobin expression patterns of newborn rats compared to E15.5 fetuses

***Protein name***	***Protein ID***	***T-value***	***P-value***	***Increasing (↑) / Decreasing (↓)***
Hbb-b1 Zero beta-1 globin	IPI00207146	2.4	0.05-0.1	↑
Hbb 16 kDa protein	IPI00951116	1	0.2-0.4	↑
Hemoglobin subunit alpha-1\2	IPI00205036	−1.9	0.1-0.2	↓
Hbb Hemoglobin subunit beta-2	IPI00231192	−2.1	0.1-0.2	↓
Hbz hemoglobin	IPI00421293	−3.5	<0.05	↓
Hbb Hemoglobin subunit beta-1	IPI00230897	−4.4	<0.05	↓
Hbe1 RCG39817	IPI00212478	−9.5	<0.05	↓
Hbg1 RCG39434	IPI00212481	−14.5	<0.05	↓

Changes in levels of hemoglobin subunits may be correlated with the biological process that occurs during development. It has been reported that the fetal hemoglobin subunit gamma has a higher oxygen affinity than hemoglobin beta [29]. This lower affinity allows the maternal hemoglobin beta to release oxygen and readily transfer its oxygen to the fetal hemoglobin subunit gamma, which allows newborns to utilize oxygen more efficiently.

### Comparison of apolipoproteins

Twelve apolipoproteins were identified in our screen, but only Apo H (IPI00778633.1) exhibited a significant increase (p < 0.05), while Apo C-II, Apo C-IV, and Apo F had a slight increase. Other Apo proteins exhibited a significant decrease (p < 0.05; Table 3). Importantly, this is the first study to find changes in the apolipoprotein expression pattern during development.

**Table 3:** Apolipoprotein expression patterns of newborn rats compared to E15.5 fetuses

***Protein name***	***Protein ID***	***T-value***	***P-value***	***Increasing (↑) / Decreasing (↓)***
Apolipoprotein H	IPI00778633	62.9	<0.05	↑
Isoform 1 of Apolipoprotein C-IV	IPI00191952	1	0.2-0.4	↑
Similar to apolipoprotein F-like	IPI00199713	1	0.2-0.4	↑
Apolipoprotein C-II	IPI00194583	0.9	>0.5	↑
Apolipoprotein A-I	IPI00197703	−3.5	<0.05	↓
Apolipoprotein E	IPI00190701	−3.5	<0.05	↓
Apolipoprotein M	IPI00207275	−3.6	<0.05	↓
Apolipoprotein A-IV	IPI00324272	−5.8	<0.05	↓
Cllusterin(apolipoprotein J)	IPI00198667	−6.7	<0.05	↓
Apolipoprotein H	IPI00195241	−7.2	<0.05	↓
Apolipoprotein A-II	IPI00197700	−9	<0.05	↓
Apolipoprotein B-100	IPI00554264	−12.1	<0.05	↓

Given the effect of hormones on the expression of Apo A-I , Apo A-IV, and Apo E [30], we hypothesized that the developmental patterns of lipometabolism proteins might be caused by late fetal stage hormone release during the maturation of the endocrine system, including the pituitary, thyroid, adrenal cortex, and p cells of the pancreas. These apolipoproteins have been reported to be involved in the transport of lipids, act as cofactors for enzymes of lipid metabolism, or maintain the structure of the lipoprotein particles [31]. Therefore, the lipometabolic functions of serum were expected undergo substantial changes during this development period.

### Comparison of complement proteins

Complement acts as a rapid and efficient immune surveillance system and contributes substantially to physiologic homeostasis by eliminating cellular debris and infectious microbes [32]. In our study, we found that the complement system exhibited a significant change between E15.5 fetuses and newborn rats (Table 4). Ten of the eleven complement factors identified increased, with four having a significant increase (p < 0.05), and only one slightly decreased. These findings were consistent with the previous study by Stabile et al., which showed that C3, C4, and Factor H had the same change during human fetal serum development [33]. For instance, C3, which plays a central role in the activation of both classical and alternative complement pathways, exhibited a significant increase of more than 10-fold in this study. It has been reported that serum levels of complement rise in newborns between birth to the first year of life [34,35], and therefore we speculate that serum levels of most complement proteins might rise between the embryonic period and infancy.

**Table 4:** Complement expression patterns of newborn rats compared to E15.5 fetuses

***Protein name***	***Protein ID***	***T-value***	***P-value***	***Increasing (↑) / Decreasing (↓)***
Complement C3 (Fragment)	IPI00480639	17.9	<0.05	↑
Complement inhibitory factor H	IPI00208659	3.7	<0.05	↑
Complement component 5	IPI00368550	3.5	<0.05	↑
Complement C4 precursor	IPI00213036	3.4	<0.05	↑
Complement factor B	IPI00382185	2.1	0.1-0.2	↑
Complement C1q subcomponent subunit C	IPI00215299	2	0.1-.2	↑
Complement factor I	IPI00204451	1.4	0.2-0.4	↑
Complement C1q subcomponent subunit B	IPI00215297	1	0.2-0.4	↑
Complement C1q subcomponent subunit A	IPI00215296	1	0.2-0.4	↑
Complement C2	IPI00194044	1	0.2-0.4	↑
Complement component C6	IPI00331776	−0.6	>0.5	↓

These results suggested that the complement system was strengthened during fetal development, which would allow the newborn rats to be more adaptive to the extrauterine environment. These results were in agreement with those obtained from the gene ontology (GO) annotation, in which significantly over-represented GO biological process terms were found for a set of significantly increased serum proteins, including those involved in the acute-phase response, acute inflammatory response, inflammatory response, defense response, response to wounding, and regulation response to external stimulus (Additional file 3: Figure S1).

**Figure 3:**
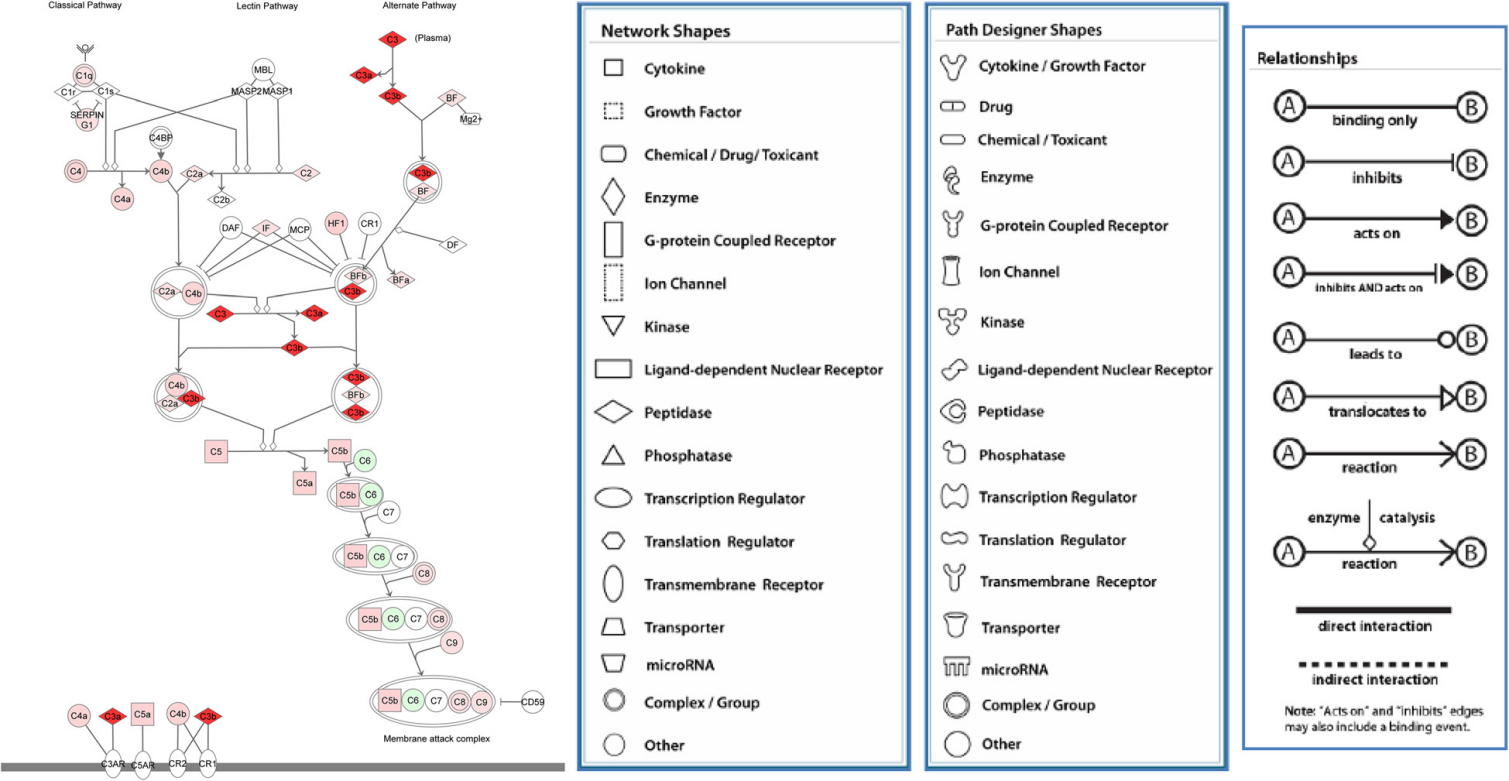
**Ingenuity pathway analysis (IPA) for complement proteins.** A complement system pathway generated by IPA. Proteins with increased expression are marked in red proteins with decreased expression are marked in green. The IPA legend is shown in Additional file 5.

Ingenuity Pathway Analysis (IPA) software was used to systematically visualize the complement proteins involved in the signal pathway (Figure 3). The changes of different complement proteins acting in different positions of the signaling pathway are also shown in Figure 3.

### Comparison of enzymes and enzyme regulators

In the GO annotations, 180 of all the identified proteins were annotated as enzymes or enzyme regulator-related. Of these, 7 enzymes and 14 enzyme regulators were significantly increased and 11 and 8 were significantly decreased, respectively, in newborn rats compared to E15.5 fetuses (p < 0.05; Tables 5 and 6). Moreover, 11 of 14 proteins with increased expression and 6 of 8 proteins with decreased expression were annotated as enzyme inhibitors. A high proportion of enzyme inhibitors was an interesting physiological phenomenon, and protease inhibitors might protect the fetus from proteases released from growing cells [36]. Moreover, changes in enzyme and enzyme regulator expression may be caused by organ maturation and the biological processes occurring in organs may exhibit a large change.

**Table 5:** Enzymes with significantly altered expression in newborn rats compared to E15.5 fetuses (P < 0.05)

***Protein name***	***Protein ID***	***T-value***	***Increasing (↑) / Decreasing (↓)***
Carboxypeptidase N	IPI00192657	10.5	↑
F2 Prothrombin (Fragment)	IPI00189981	7.2	↑
Hepatocyte growth factor activator	IPI00364125	5	↑
Hp Isoform 1 of Haptoglobin	IPI00382202	4.9	↑
Plasminogen	IPI00206780	4.9	↑
Pragmin tyrosine-protein kinase SgK223	IPI00358819	4.6	↑
Smarcad1 Uncharacterized protein	IPI00765483	3.3	↑
L-lactate dehydrogenase A chain	IPI00197711	−3.2	↓
Cathepsin B preproprotein	IPI00212811	−3.7	↓
Peroxiredoxin-2	IPI00201561	−4.4	↓
Coagulation factor X	IPI00206786	−4.4	↓
Tuba1b Uncharacterized protein	IPI00339167	−4.9	↓
Proprotein convertase subtilisin\kexin type 9	IPI00396889	−6.2	↓
Fructose-bisphosphate aldolase A	IPI00195851	−8	↓
pyruvate kinase-like isoform 2	IPI00339197	−8.2	↓
Tubulin beta-2B chain	IPI00195673	−11.7	↓
Nucleoside diphosphate kinase B	IPI00194404	−12.9	↓
Glutathione peroxidase 3	IPI00476458	−15.5	↓

**Table 6:** Enzyme regulators with significantly altered expression in newborn rats compared to E15.5 fetuses (P < 0.05)

***Protein name***	***Protein ID***	***T-value***	***Increasing (↑) / Decreasing (↓)***
Apoh Apolipoprotein H	IPI00778633	62.9	↑
C3 Complement C3 (Fragment)	IPI00480639	17.9	↑
Serpina1 Alpha-1-antiproteinase *	IPI00324019	16.6	↑
Itih3 Uncharacterized protein *	IPI00326984	8.6	↑
A2m Alpha-2-macroglobulin *	IPI00392886	8.2	↑
Serpina3k Serine protease inhibitor A3K *	IPI00200593	4.9	↑
Kng1l1 T-kininogen 2 *	IPI00679245	4.8	↑
Itih4 inter-alpha-inhibitor H4 heavy chain *	IPI00188541	4.7	↑
Uncharacterized protein *	IPI00958555	3.8	↑
C4b complement C4 precursor	IPI00213036	3.4	↑
Serpina3n Serpina3n-like protein *	IPI00211075	3.2	↑
Pzp Alpha-1-macroglobulin *	IPI00326140	3.2	↑
Kng1;Kng2 Isoform HMW of Kininogen-1 *	IPI00187799	3.2	↑
Ahsg Alpha-2-HS-glycoprotein *	IPI00327469	3.2	↑
Serpinf1 Serine (Or cysteine) peptidase inhibitor *	IPI00199670	−3.3	↓
Apoe Apolipoprotein E	IPI00190701	−3.5	↓
Apoa1 Apolipoprotein A-I *	IPI00197703	−3.5	↓
Serpinf2 Serine (Or cysteine) peptidase inhibitor *	IPI00199695	−3.8	↓
Serpina6 Corticosteroid-binding globulin *	IPI00210824	−4.1	↓
Cst3 Cystatin-C *	IPI00231801	−6.6	↓
Apoh Apolipoprotein H	IPI00195241	−7.2	↓
Apoa2 Apolipoprotein A-II *	IPI00197700	−9	↓

Note: Terms marked with an asterisk (*) represent enzyme inhibitors.

### Comparison of other differentially expressed proteins

Other proteins with significantly altered expression levels with one or more known functions annotated in the UniProt database were listed in Table 7. The proteins with unknown function that had significantly altered expression levels between the two groups were shown in Table 8. Although the functions of these proteins are currently unknown, they changed quantitatively, which indicates that these proteins might be key molecules involved in development.

**Table 7:** Other proteins with known functions that had significantly altered expression in newborn rats compared to E15.5 fetuses changed proteins (P < 0.05)

***Protein name***	***Protein ID***	***T-value***	***Increasing (↑) / Decreasing (↓)***	***Functions (noted in uniprot database)***
Isoform 1 of Serotransferrin	IPI00679202	57.1	↑	iron binding transport
Zdhhc5 Uncharacterized protein	IPI00193933	22.9	↑	contains 1 DHHC-type zinc finger
CD320 antigen-like	IPI00365976	6.2	↑	augmenting the proliferation of PC precursors generated by IL-10
Afamin	IPI00207668	5.5	↑	vitamin E binding protein
hypothetical protein	IPI00781081	3.9	↑	binding various heavy metals
Myoblast determination protein 1	IPI00205974	3.7	↑	myogenic factor
Gc Vitamin D-binding protein	IPI00194097	3.2	↑	carrying the vitamin D sterols, preventing polymerization of actin
Heat shock protein HSP 90-beta	IPI00471584	−3.4	↓	molecular chaperone in cell cycle control and signal transduction
Fga protein	IPI00202651	−3.4	↓	yielding monomers, acting as a cofactor in platelet aggregation
Ywhaz 14-3-3 protein zeta\delta	IPI00324893	−4	↓	adapter protein in the regulation of signaling pathways
actin	IPI00189819	−4.3	↓	involved in cell motility
Collagen alpha-1(III) chain	IPI00366944	−5.3	↓	soft connective tissues along with type I collagen
Collagen alpha-2(I) chain	IPI00188921	−5.9	↓	member of group I collagen
Col1a1 Rat alpha-1 type I collagen	IPI00188909	−6	↓	member of group I collagen
Scn5a 228 kDa protein	IPI00231550	−6.7	↓	mediating the voltage-dependent sodium ion permeability
Platelet factor 4	IPI00206634	−7.6	↓	chemotactic for neutrophils and monocytes, Inhibits endothelial cell proliferation
heat shock cognate 71 kDa protein-like	IPI00207355	−7.7	↓	cooperation with other chaperones
Cadherin-5	IPI00768626	−9.8	↓	calcium dependent cell adhesion proteins
Isoform Gamma-A of Fibrinogen	IPI00190759	−10	↓	yielding monomers, acting as a cofactor in platelet aggregation
growth factor-binding protein 4	IPI00206239	−11.9	↓	alter the interaction of IGFs with their cell surface receptors

**Table 8:** Proteins with unknown functions that had significantly altered expression in newborn rats compared to E15.5 fetuses changed proteins (P < 0.05)

***Protein name***	***Protein ID***	***T-value***	***Increasing (↑) / Decreasing (↓)***	***Functions (Gene Ontology)***
Megf11 Protein	IPI00765428	8.6	↑	
Igk protein-like isoform 2	IPI00568389	7.2	↑	
Hrc 87 kDa protein	IPI00331867	5.7	↑	histidine-rich calcium binding protein
Vtn Aa1018	IPI00210120	5.2	↑	polysaccharide binding, scavenger receptor activity
LRRGT00161	IPI00655254	4.6	↑	ferric iron binding
Thbs4 106 kDa protein	IPI00197194	4.4	↑	structural molecule activity
rCG47051-like	IPI00958198	4	↑	
Lrp1 prolow-density lipoprotein receptor-related protein 1	IPI00369995	4	↑	protease binding
Itgb3 Integrin beta	IPI00198695	3.9	↑	peptide binding, receptor activity
- 8 kDa protein	IPI00782171	3.5	↑	
Ppp1r12b Uncharacterized protein	IPI00371976	3.5	↑	
Ig lambda-2 chain C region	IPI00370486	3.5	↑	antigen binding
mCG147639-like	IPI00557598	3.4	↑	
Cd97 90 kDa protein	IPI00365168	3	↑	G-protein coupled receptor activity, calcium ion binding
Cfp Properdin factor	IPI00365896	2.9	↑	
Ltbp4 Uncharacterized protein	IPI00204867	2.9	↑	calcium ion binding
Uncharacterized protein	IPI00950846	−2.8	↓	nucleic acid binding, nucleotide binding
Eef1b2	IPI00372520	−3.4	↓	translation elongation factor activity
Lumican	IPI00206403	−3.8	↓	
Uncharacterized protein	IPI00948226	−3.9	↓	
A030009H04Rik	IPI00201907	−4.1	↓	zinc ion binding
Collagen	IPI00189470	−4.5	↓	
Rps5 protein	IPI00886474	−4.5	↓	ribonucleoprotein
histone cluster 1	IPI00188688	−5	↓	DNA binding
Uncharacterized protein	IPI00952007	−5.6	↓	
Fibrillin-2	IPI00204009	−5.7	↓	extracellular matrix structural constituent;calcium ion binding)
Fbln1 protein	IPI00557007	−6.6	↓	calcium ion binding
Postn Uncharacterized protein	IPI00190088	−6.8	↓	
Cadherin 11	IPI00211883	−9.7	↓	calcium ion binding
Uncharacterized protein	IPI00778692	−15.9	↓	
Tf 107 kDa protein	IPI00196656	−26.6	↓	GTP binding,ferric iron binding, ubiquitin protein ligase binding
Talin-1	IPI00362014	−30.6	↓	actin binding

Note: Proteins were annotated in the UniProt database as uncharacterized proteins.

### Proteome variations in serum from individual littermates in the E15.5 fetus and newborn rat groups

**Figure 4:**
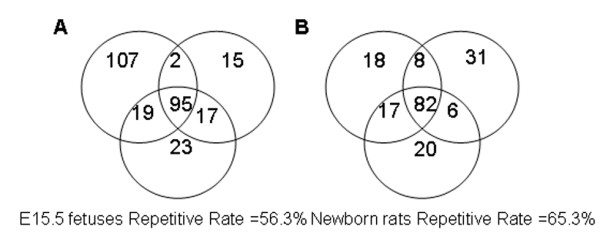
**Repetitiveness of individual specimens.A** and **B** indicate the repetitiveness of proteins identified in three E15.5 fetuses (**A**) and three newborn rats (**B**), respectively. Each circle in A and B represents an individual sample. The numbers represent the number of shared proteins in the respective overlapping areas. The repetitiveness of the individual specimens was evaluated based on the repetitive rate of protein identification (E15.5 fetuses, Repetitive Rate = 56.3%; newborn rats, Repetitive Rate = 65.3%).

The variations in the serum proteome between littermates were studied based on 1D LC-MS/MS. Three individual serum samples each from E15.5 fetuses and newborn rats were analyzed in duplicate. The technical variability of the LC-MS/MS method was investigated using a triplicate analysis of pooled samples generated by pooling six individual serum samples from each stage. The repetitiveness of individual serum protein identifications for E15.5 fetuses and newborn rats was also calculated for each sample (Figure 4). There was a remarkable difference in the repetitive rate of individual samples (E15.5 fetuses = 56.3%; newborn rats = 65.3%) compared to the pooled samples (E15.5 fetuses = 68.4%; newborn rats = 78.7%; data not shown). Therefore, differences in the repetitive rates between the individual and pooled samples are most likely due to littermate variations.

To investigate variation between littermates, the coefficient of variation (CV) of spectral counts for each protein in the three individual samples and in the triplicate analysis of pooled samples were calculated for both E15.5 fetus and newborn rat samples, respectively. Considering that proteins with low abundance have larger variation in MS identification, the CV ratio between individual and pooled samples was calculated only for the proteins with average spectra counts greater than six. We even identified some proteins with medium and high abundance that had larger CV values in the three individual samples than in the triplicate analysis of the pooled samples (Additional file 4: Figure S2), which indicates that these proteins exhibit true biological variation among littermates.

Some proteins with differential expression between littermates are noteworthy, such as Apo H, Fatty Acid Synthase, Hemoglobin subunit alpha-1\2, Peroxiredoxin-2, and Elongation Factor 2 in E15.5 fetuses as well as Complement 3, Inter-alpha-Trypsin Inhibitor and Thrombospondin 1 in newborn rats. Notably, Complement 3, Inter-alpha-Trypsin Inhibitor, Apo H, and Peroxiredoxin-2 are important molecules for the regulation of body homeostasis, and Complement 3 is related to the immune system. However, other proteins showed minimal variation, such as Kininogen 1, IgG-2a, and Serotransferrin in E15.5 fetuses as well as Complement Inhibitor Factor H and IgG-2a in newborn rats. Therefore, these results suggest that even in littermates with a similar genetic background, some proteins in the serum have a substantial variation while others do not.

## Conclusions

To the best of our knowledge, this is the first study to analyze serum proteome changes during development using LC-MS/MS. The serum proteomes of newborn rats and E15.5 fetuses were compared. We found that expression patterns of hemoglobin subunits were different in newborn rats compared to E15.5 fetuses, whereby most had decreased expression. The majority of apolipoproteins also significantly decreased, and almost all identified complement molecules increased. In addition the levels of several highly abundant serum proteins varied between littermates in these two developmental stages.

## Materials and methods

### Sample preparation

This study was approved by the Institute of Basic Medical Sciences Animal Ethics Committee at the Peking Union Medical College (Animal Welfare Assurance Number: # A5518-01). Rats were caged and handled under ethical conditions, according to international rules of animal care specified in the International Animal Welfare Recommendations. Sprague–Dawley rats weighing 250 g were purchased from the Huafukang Biotechnical Company (Beijing, China). The day at which spermatozoa were present in the vaginal smear was recorded as half a day of gestation. Blood of E15.5 fetuses was obtained from the umbilical cord, and blood of newborn rats was obtained from the jugular vein, as previously described [9]. To avoid potential contamination, the umbilical cord was first washed with 0.9% NaCl solution for E15.5 fetuses, and the first drop of blood was discarded for newborn rats. In all cases, the blood was allowed to clot for approximately 4 h in silicone centrifuge tubes at 4°C. The clotted material was removed by centrifugation at 1000 g for 15 min. The resulting serum was then centrifuged at 12000 g for 15 min at 4°C to remove any remaining cell debris. The serum supernatant was collected and frozen at −80°C [37]. An additional two pooled samples were prepared by mixing an equal amount of protein from 6 different E15.5 fetuses and newborn rats, respectively.

### One-dimensional SDS-PAGE analysis

The extracted proteins (20 μg) was dissolved by mixing the samples with loading buffer, boiled for 5 min, and loaded onto a 10% SDS-PAGE. After separation, the proteins were stained with Coomassie Brilliant Blue.

### Mass spectrometry (MS) analysis

In our study, triplicate analyses of LC-MS/MS were performed on the pooled specimens for E15.5 fetuses and newborn rats, respectively. Single or replicate analyses of LC-MS/MS were performed on individual specimens. Proteins were reduced, alkylated, and trypsin digested as previously described [38]. The tryptic peptides were desalted by solid-phase extraction (Oasis column; Waters, Inc, Milford, Massachusetts, USA) and dried by vacuum evaporation. The dried peptides were re-dissolved in an aqueous solution containing 0.1% formic acid [39]. For LC-MS/MS analyses, the peptides were sequentially loaded onto a trap column (Michrom peptide Captrap, MW 0.5-50 kD, 0.5 × 2 mm; Michrom Bioresources, Inc, FOB Auburn, CA, USA) at a flow rate of 20 μL/min with mobile phase (0.1% formic acid, 99.9% water). The trap column effluent was then transferred to a reversed-phase microcapillary column (0.1 × 150 mm, packed with Magic C18, 5 μm, 200 Å; Michrom Bioresources, Inc, FOB Auburn, CA, USA) in an Eksigent system (AB, Inc, Framingham, Massachusetts, USA). Separation of the peptides was performed at a flow rate of 500 nL /min and coupled to an online analysis by tandem MS using LTQ Orbitrap Velos (Thermo Fisher Scientific, San Jose, USA). The elution gradient for the reverse column was changed from 95% mobile phase (0.1% formic acid, 99.9% water) to 40% mobile phase (0.1% formic acid, 99.9% acetonitrile). The elution time was 150 min, except for the analysis of individual variation, where it was 100 min. The MS was programmed to acquire data in a data-dependent mode [40]. For the pooled and individual samples used for individual variation analysis, all survey scans were acquired in the Orbitrap mass analyzer and the lock mass option was enabled for the 445.120025 ion [41]. The MS survey scan was obtained for the m/z range 300–2000 amu with a resolution of 30000, followed by data-dependent MS/MS scans (isolation width of 3 m/z, dynamic exclusion for 0.5 min), and the twenty most intense ions were fragmented by higher energy collision dissociation (HCD) in the collision cell (normalized collision energy of 40%; the activation time was set to 0.1 s) and detected in the Orbitrap analyzer at 7500 resolution. For the six individual specimens used for quantification analysis, MS survey scans were acquired in the Orbitrap analyzer at 60000 resolution and MS/MS were analyzed in LTQ analyzer. The twenty most intense ions were fragmented in the ion trap by collision-induced dissociation with a normalized collision energy of 35%, activation q value 0.25, and activation time of 10 ms.

### Protein identification

Peptide identification was performed using the SEQUEST algorithm-based Bioworks 3.3.1 (Thermo Scientific, Inc, San Jose, USA) to search the rat IPI 3.82 protein sequence database. The search parameters were set as follows: precursor mass tolerance, 5 ppm; fragment mass tolerance, 0.5 amu in LTQ detector and 10 mmu in Orbitrap detector; tryptic cleavages at only a lysine or arginine with up to two missed cleavage sites allowed; and a static modification of +57.02150 amu on cysteine. The search results were further processed by the Trans-Proteomic Pipeline (TPP) software (Developed by the Institute for Systems Biology (ISB) in the Seattle Proteome Center.) and the SEQUEST results were validated by PeptideProphet [42], which also calculates the probability of peptide identification. ProteinProphet [43] was then applied to assign each peptide to a protein and calculate the probability of protein identification. The probability of protein identification was calculated based on the peptide probability and the SEQUEST Xcorr score [43]. Only protein identifications with a probability > 0.95 were considered for further analysis, as this cutoff resulted in a calculated FDR lower than 1%.

### Individual variation

Repetitiveness of samples from E15.5 fetuses and newborn rats was calculated using the formula: repetitive rate = the number of common identified proteins / the average number of identified proteins × 100%. To investigate the variation between individual littermates, the coefficient of variation (CV) was calculated using the formula: CV = the standard deviation of the spectral counts/ the average spectral counts × 100%. The triplicate analysis of pooled specimens in the same stage based on LC-MS/MS was used as a technical control. Each protein’s CV ratio between individual and pooled samples was used to reflect the variation of this protein in individual specimens. However, the variation of low abundant proteins was generally large due to the random sampling nature of the mass spectrometry. Therefore, we calculated the CV ratios of the proteins that had average spectral counts greater than six in both the pooled and individual samples.

### Quantification

The relative protein abundance was estimated based on spectral counts (SC) of each given protein [44]. To reduce the bias of the peptide amount loaded in each experiment, the SC were normalized for each protein by dividing the SC by the total SC identified in each run [45]. A two-tailed t-test was used to analyze significant differences in identified proteins between two different specimens (p < 0.05) [46].

### Western blots

Western blots were performed to validate the changes in Complement 3, Hemoglobin epsilon 1, and Apolipoprotein B in five E15.5 fetus serum samples and five newborn rat serum samples. From these samples, 30 μg proteins were loaded and separated on 12% SDS-PAGE. A mini Trans-Blot Cell system (Bio-Rad Laboratories Co., Ltd. Shanghai, China) was used to perform the transfer to a nitrocellulose membrane following the manufacturer’s protocols. After blocking with 5% non-fat milk, membranes were probed with chicken anti-Apolipoprotein B (ab117317, Abcam Hong Kong Ltd, HK), rabbit anti-HBE1 (12361-1-AP, Proteintech Group, Inc, Chicago, USA), and rat anti-C3 (CL7334AP, Cedarlane, Canada). Secondary antibodies were purchased from Zhongshan Goldenbrige Biotechnology Company (Beijing, China) and EarthOx (San Francisco, CA 94121, USA). The protein bands were detected using the Enlight Western Blot kit (Engreen Biosystem Co, Ltd. Beijing, China). The densitometry of the bands was calculated using ImageJ, which is a public domain Java image processing and analysis program inspired by NIH Image for the Macintosh (Obtained from http://imagej.nih.gov/ij/docs/guide). A t-test was performed to analyze significant differences between different bands.

### Enrichment analysis of gene ontology (GO) categories

The identified proteins were functionally categorized based on universal GO annotation terms [47] using the Biological Networks Gene Ontology (BiNGO) program package [48]. For enrichment analysis, we constructed a test dataset consisting of the proteins identified that had significant changes as well as a reference set of GO annotation for all identified serum proteins. As per instructions on the BiNGO webpage, the custom GO annotation for the reference set was created by extracting the GO annotations available from the EBI GOA rat 2.0 release [49], which contains annotations for 27746 proteins compiled from different sources. The analysis was performed using a “hyper-geometric test”, and all GO terms that were significant (P < 0.001 after correcting for multiple term testing by Benjamini and Hochberg false discovery rate corrections) were selected as being over-represented and under-represented.

### Ingenuity pathway analysis (IPA)

IPA was used to identify gene networks according to biological functions and/or diseases in the Ingenuity Pathways Knowledge Base (Ingenuity Systems, Redwood City, CA). IPI numbers of identified proteins were the screened in the Ingenuity Pathways Analysis (IPA) Knowledge Base.

## Competing interests

The authors declare that they have no competing interests.

## Authors’ contributions

LW and YG were responsible for planning and designing the study. LW and LZ collected samples. CS and DZ developed the protocols. Lilong Wei and Lulu Jia performed the experiments. Sucan Ma and Wei Sun performed the data analysis. Lilong Wei wrote the manuscript. YG helped to revise the manuscript. All authors read and approved the final manuscript.

## Supplementary Material

Additional file 1 **Table S1.** Comparison details. Click here for file

Additional file 2 **Table S2.** Densitometries of the bands in Figure 2. Click here for file

Additional file 3 **Figure S1.** Biological Process overrepresented. Significantly overrepresented GO biological process terms for the set of significantly increased serum proteins. In total, 552 and 590 proteins were linked to at least one annotation term within the GO molecular function and biological process categories, respectively. The set of the significantly increased proteins was compared to all of the identified serum proteins. Proteins with P < 0.001 are shown. The ratio shown is the number of significantly increased proteins and all identified proteins to each GO term divided by the number of increased and all serum proteins linked to at least one annotation term within the indicated GO biological process and molecular function categories. GO, Gene Ontology; IPI, International Protein Index. Click here for file

Additional file 4 **Figure S2.** Variations of serum high abundant proteins, To investigate the variation between individual littermates, the coefficient of variation (CV) was calculated using the formula: CV = the standard deviation of the spectral counts/ the average spectral counts × 100%. The proteins’ CV ratios between individual and pooled samples were plotted against the average spectral counts of the proteins in the triplicate analysis of the pooled samples for the E15.5 fetuses (A) and newborn rats (B) specimen respectively. Only proteins with average spectral counts more than six both in pooled and individual samples were analyzed. Click here for file

Additional file 5 Protein identifications in MS. Click here for file
