# Supplementary figures and images for: A comparison of E15.5 fetus and newborn rat serum proteomes
Source: Proteome Sci. 2012 Nov 7;10:64. doi: 10.1186/1477-5956-10-64 (PMC3583134; doi:10.1186/1477-5956-10-64)

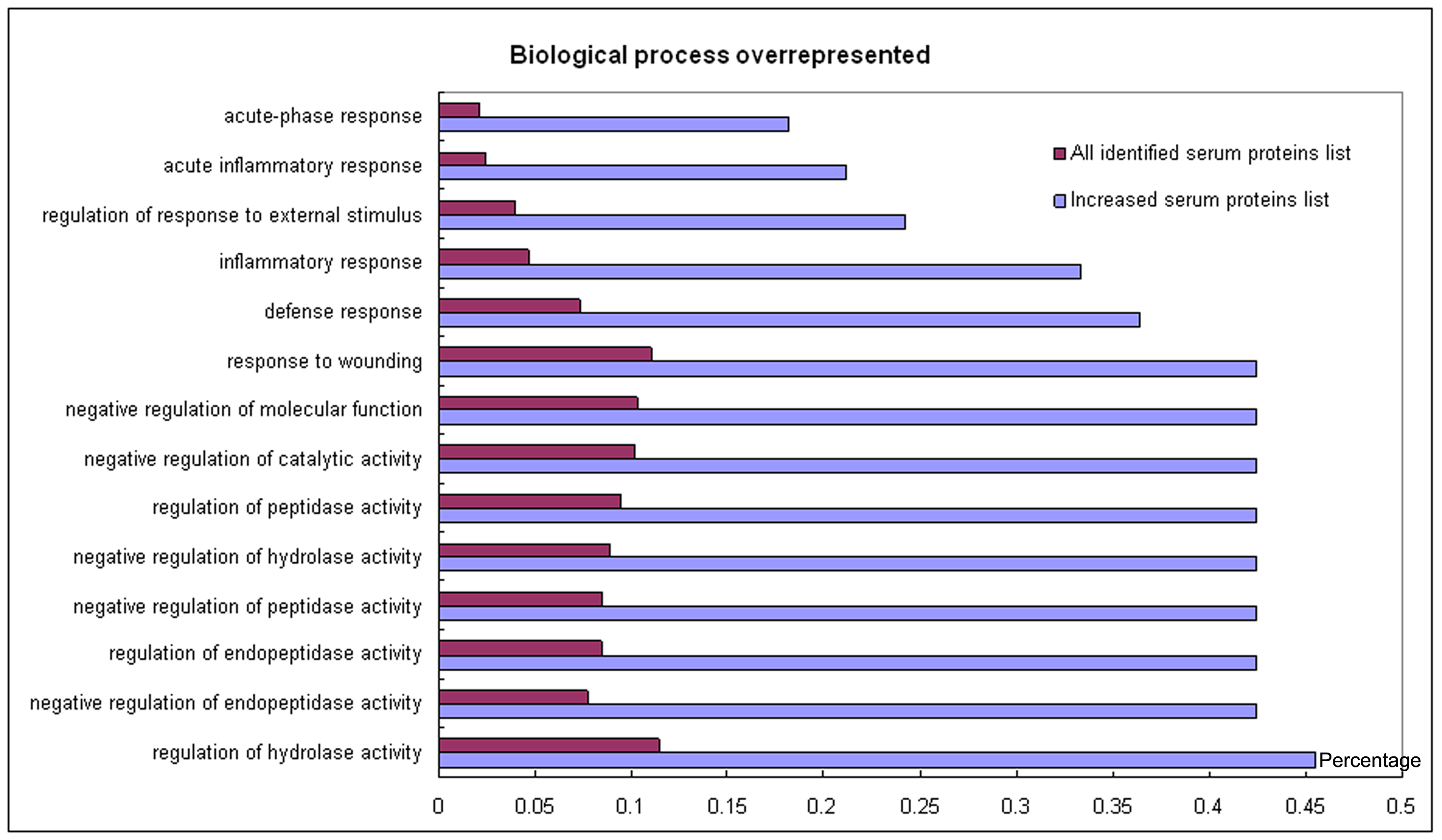

Supplement: Additional file 3 — Figure S1. Biological Process overrepresented. Significantly overrepresented GO biological process terms for the set of significantly increased serum proteins. In total, 552 and 590 proteins were linked to at least one annotation term within the GO molecular function and biological process categories, respectively. The set of the significantly increased proteins was compared to all of the identified serum proteins. Proteins with P < 0.001 are shown. The ratio shown is the number of significantly increased proteins and all identified proteins to each GO term divided by the number of increased and all serum proteins linked to at least one annotation term within the indicated GO biological process and molecular function categories. GO, Gene Ontology; IPI, International Protein Index. [file 1477-5956-10-64-S3.tiff]

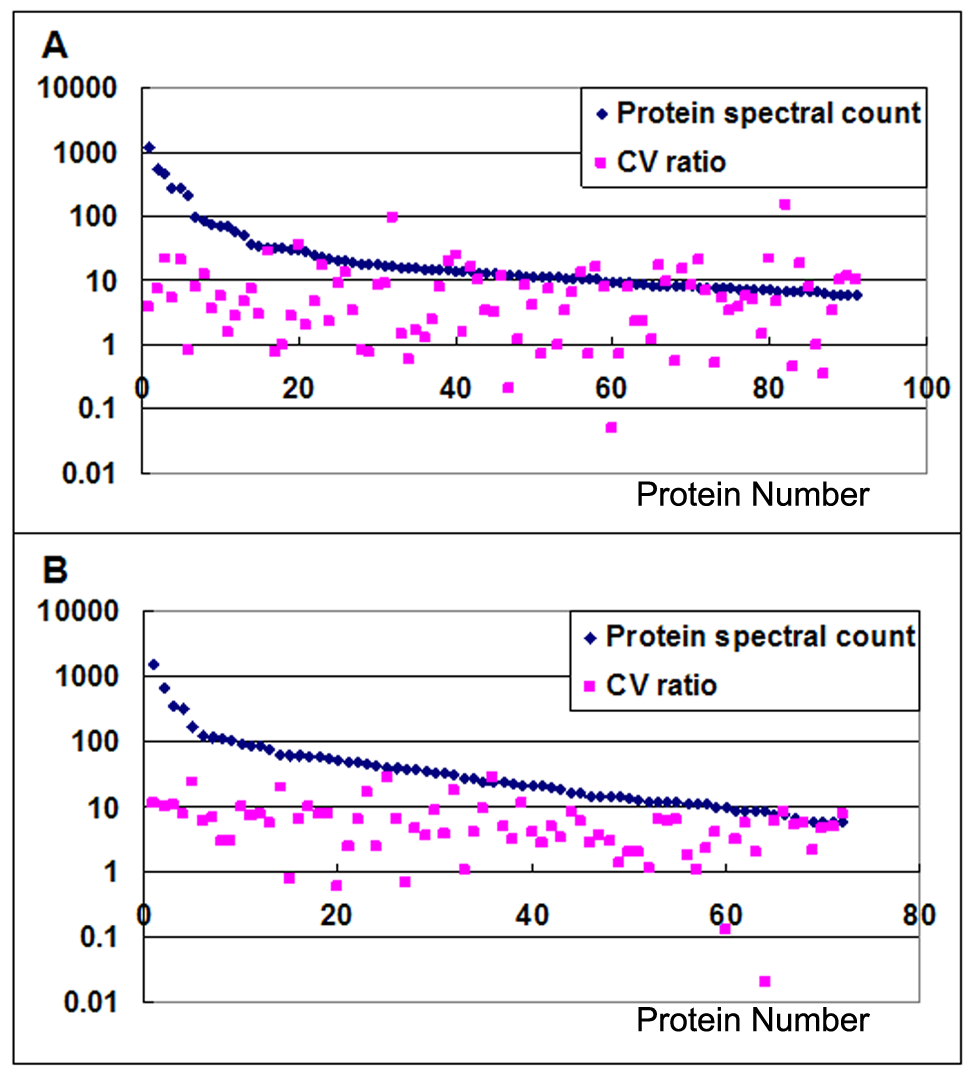

Supplement: Additional file 4 — Figure S2. Variations of serum high abundant proteins, To investigate the variation between individual littermates, the coefficient of variation (CV) was calculated using the formula: CV = the standard deviation of the spectral counts/ the average spectral counts × 100%. The proteins’ CV ratios between individual and pooled samples were plotted against the average spectral counts of the proteins in the triplicate analysis of the pooled samples for the E15.5 fetuses (A) and newborn rats (B) specimen respectively. Only proteins with average spectral counts more than six both in pooled and individual samples were analyzed. [file 1477-5956-10-64-S4.tiff]
